# Operational Feasibility of Hospital-Based Cancer Registries in Low- and Middle-Income Countries: A Systematic Review

**DOI:** 10.7759/cureus.42126

**Published:** 2023-07-19

**Authors:** Shubharanjan Jena, Krushna Chandra Sahoo, Kajal Samantaray, Nancy Satpathy, Venkatarao Epari

**Affiliations:** 1 Community Medicine, Siksha 'O' Anusandhan Deemed to be University Institute of Medical Sciences and SUM Hospital, Bhubaneswar, IND; 2 Public Health, Indian Council of Medical Research (ICMR) - Regional Medical Research Center, Bhubaneswar, IND; 3 Public Health, Institute of Public Health, Bengaluru, Bengaluru, IND

**Keywords:** developing countries, health system strengthening, cancer surveillance, digital health, cancer registry

## Abstract

Cancer registration is crucial for any country's cancer surveillance and management program. However, there is a lack of systematic evidence on the operational feasibility of hospital-based cancer registries (HBCRs) in low- and middle-income countries (LMICs). We systematically reviewed and described the challenges and prospects of HBCRs in LMICs. We reported the study according to the Preferred Reporting Items for Systematic Review and Meta-Analysis Protocols (PRISMA-P) guidelines. Electronic databases such as MEDLINE, EMBASE, Web of Science, ProQuest, and CINAHL were searched. Peer-reviewed studies published between January 1, 2000 and June 30, 2021 were included. We used thematic analysis to synthesize the findings discussing barriers and enablers of HBCRs. Thirteen studies were eligible for the analysis after eliminating duplicates, screening of title and abstract, and full-text review. The determinants, registry functionality, data management and abstraction, data security, data quality, organizational readiness, and perception of registry staff influence the implementation of HBCRs. In LMICs, many registries lacked functional documentation and data management systems due to a shortage of skilled professionals. However, in many instances, physicians and patients communicated via digital media, which helped obtain accurate data. The HBCR completeness rate was high in Ethiopia, China, Thailand, and Tanzania. Qualification and capacity building of the data managers was linked to the completeness and accuracy of the registry data, which led to sustainability. In addition, a few registries implemented new worksheets to enhance documentation. This review highlights the need for additional digitalization of the cancer registry to improve its functionality, completeness, follow-up, and output. Further, physicians and data managers require regular training to address cancer registry completeness and reduce errors.

## Introduction and background

Cancer is the world's second-largest cause of death, killing an estimated 9.6 million people in 2018 [1]. Every year, over eight million people die from cancer in low- and middle-income countries (LMICs) due to poor-quality care [2]. In 2017, the WHO recommended that national governments establish a global action plan for non-communicable disease prevention and control; the 2030 United Nations Agenda was to reduce cancer-related premature death [3]. Cancer patients in LMICs have a poor prognosis due to variables such as late presentation, insufficient cancer knowledge, and unequal distribution of cancer care facilities [4]. By 2030, LMICs will be responsible for about 75% of all cancer-related morbidity and mortality, with one in eight people developing the disease during their lifetime [5]. Cancer registration is fundamental to cancer surveillance and cancer control programs in any nation [3]. The cancer registry continuously gathers data on the occurrence and characteristics of reportable neoplasms and provides accurate data on cancer incidence and survival [6]. Hospital-based cancer registries (HBCR) and population-based cancer registries (PBCR) are the two types of cancer registries. The HBCR gathers data on cancer patients who visit a particular hospital, whereas the PBCR collects data on newly diagnosed cancer patients in a well-defined community [7]. Pramesh CS et al. found that only 81 out of 190 countries have nationwide coverage for cancer registries [5]. In addition, in many LMICs, site-specific cancer incidence, mortality, and stage of cancer are inadequately documented or non-existent. Only one in five countries reports cancer data of a sufficient quality to derive incidence estimates. However, while PBCR is considered the gold standard for determining cancer incidence in a specific community, implementing it is more challenging. Low PBCR coverage is mainly seen in South America (19%), Asia (15%), and Africa (13%). Further, most LMICs lack governance for effective cancer prevention, management, and control [5,8]. On the other hand, HBCR requires fewer resources [9]. HBCRs are crucial in low-resource settings when PBCRs are missing [10]. Historically, LMICs have more HBCR than PBCR [11]. Moreover, the HBCR could be an initial step toward establishing PBCRs [12]. Therefore, we systematically reviewed and described the challenges and prospects of HBCRs in LMICs.

## Review

Methods

Search Strategy and Selection Criteria

This systematic review included studies addressing operational feasibility, opportunities, and challenges related to the HBCRs in LMICs. We included LMIC countries per the latest world bank classification [13]. We included studies published from January 1, 2000 to June 30, 2021. We searched through five electronic databases: MEDLINE, EMBASE, Web of Science, ProQuest, and CINAHL. Google Scholar was searched for grey literature using key terms.

We developed a multi-faceted search approach customized for each database and a combination of text and index terms. We searched using keywords in English: "hospital-based," "medical record," and "data quality," combined with "registry," "cancer," and "tumor," "neoplasms," including all subheadings. Studies based on PBCRs, laboratory-based cancer registries, reviews, editorials, opinion articles, and dissertations were excluded. The table (Appendix 1) describes one of the search criteria used for the PubMed database.

We searched databases and entered all records into EndNote for duplicate removal; after removing duplication, we imported the records into Rayyan software for the title and abstract screening. The authors, Jena S and Samantaray K, separately screened the titles and abstracts. We resolved the disagreement with the author, Sahoo KS. The potentially eligible articles were then subjected to full-text screening by authors Jena S and Samantaray K, with other authors resolving the conflicts.

Inclusion and Exclusion Criteria

We included peer-reviewed studies discussing barriers and enablers of HBCRs publishing primary or secondary data or perspectives of stakeholders involved in the registry process in LMICs. The review was limited to articles in the English language only. A detailed summary of inclusion and exclusion criteria is presented in Table 1.

**Table 1:** Inclusion and exclusion criteria. HBCR: Hospital-based cancer registry; LMIC: Low- and middle-income countries.

Characteristics	Inclusion criteria	Exclusion criteria
Population	Hospital-based cancer registry, perspectives of stakeholders involved in the registry process.	Other than a hospital-based cancer registry
Intervention	The challenges and opportunities are associated with the implementation of hospital-based cancer registries in LMICs.	Interventions that are not specified in the inclusion criteria.
Outcome	Functionality and process of HBCR in LMIC, quality control, and HBCR sustainability-related factors	Outcomes, other than those specified in the inclusion criteria.
Study design	Quantitative, qualitative, or mixed method. Quantitative study designs included experimental and observational.	Nonempirical/Primary research including review, meta-analysis, editorial, commentary, letter to the editor, opinion paper, newspaper, protocols, and case report.
Geographic scope	LMICs	Areas other than LMICs
Time frame	January 1, 2000 to June 30, 2021	Studies published before January 2000

Data Analysis 

The first author extracted data for each article into a pre-formatted data extraction sheet using the following criteria: author, year of publication, study population, country of origin, challenges, coping strategies, preparedness, and innovativeness. We assessed the quality of selected studies using the mixed methods appraisal tool (MMAT) [14]. For data synthesis, we used the thematic analysis approach [15]. The authors thoroughly read the articles and identified the primary concept concerning the study's objective. In MAXQDA version 18.2.4, we created a coding framework for selective coding. We registered this review with the International Prospective Register of Systematic Reviews (PROSPERO), having the registration number CRD42021235574. We used the PRISMA-P checklist to report the study findings [16].

Results

**Figure 1:**
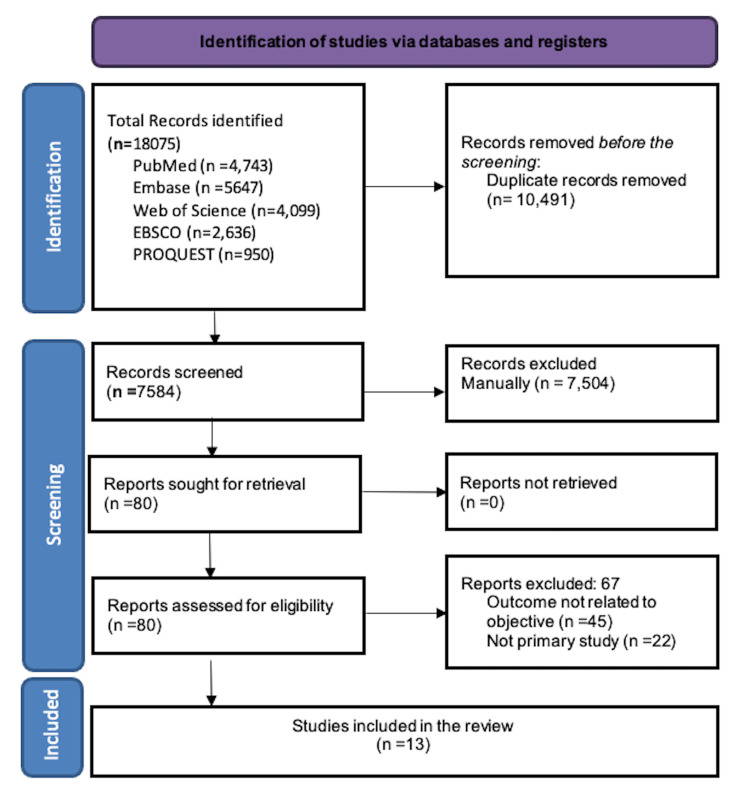
PRISMA flow diagram of selection of articles. PRISMA: Preferred Reporting Items for Systematic Review and Meta-Analysis.

A total of 18,075 records were retrieved after deleting duplicates. Of them, 7584 records were chosen for title and abstract screening, 80 for full-text review, and 13 studies (12 quantitative and one mixed-method) were finally included in the study. The PRISMA flow diagram illustrates the study selection process in Figure 1.

We reported the findings under three primary domains: functionality and process, quality control, and HBCR sustainability-related factors. The detailed characteristics of the selected studies are presented in Table 2.

**Table 2:** Characteristics of the included studies. HBCR: Hospital-based cancer registry.

Author, Year	Country	Study design/approach	Target population/study unit	Sample Size	Major domain	Outcome
Jedy-Agba EE et al. (2012) [17]	Nigeria	Quantitative cross-sectional survey	Patients	11	Sustainability	Reliability and case records
Bhatt A et al. (2017) [18]	India	Record observation and stakeholders’ interaction	Patients and staff	5	Process and challenges	Functionality, data security, and confidentiality
Ayoub L et al. (2007) [19]	Honduras	Quantitative cross-sectional survey	Leukaemia patients	652	Sustainability	Completeness, data accuracy, and obstacles
An YB et al. (2021) [20]	China	Quantitative cross-sectional survey	Tertiary centers	23	Process	Completeness and data accuracy
Buhlinger KM et al. (2021) [21]	Ethiopia	Mixed-method (descriptive-analytical)	Hospital staffs	13	Process	Comparability, completeness, and organizational readiness
Ye X et al. (2017) [22]	China	Quantitative cross-sectional survey	Patients	5080	Process	Data collection method, data consistency, and validity
Al-Haddad BJ et al. (2015) [23]	Nigeria	Quantitative cross-sectional survey	Facilities having HBCR	12	Process	Comparability, diagnostic validity, and completeness
Zullig LL et al. (2016) [24]	Tanzania	Quantitative cross-sectional survey	Patients	100	Process and opportunities	Comparability, diagnostic validity, and completeness
Sirirungreung A et al. (2018) [25]	Thailand	Quantitative cross-sectional survey	Medical Records	1	Process and challenges	Comparability, diagnostic validity, and completeness
Levy S et al. (2021) [26]	Ethiopia	Interview, secondary data	Pediatric oncology patients	27	Process and opportunities	Completeness and data accuracy
Zullig LL et al. (2013) [27]	Tanzania	Quantitative cross-sectional survey	Clinicians, nurses, and administrators	64	Process	Resources, funding, and organization motivation
Sultan F et al. (2014( [28]	Pakistan	Retrospective record review	Electronic hospital records	1	Process	Quality improvements
Banihani S et al. (2021) [29]	Jordan	Quantitative cross-sectional survey	Nurses	271	Process and challenges	Nurses' attitude towards computerization

Functionality and Process of the HBCR

Registry setting-up and its functionality: In LMICs, HBCRs play a crucial role in enhancing the healthcare system [23]. A study in India demonstrated that a cancer registry might be maintained economically by combining it with web-based tools and the training of surgeons [21]. A Nigerian study described its significance and how it might contribute to improving the health system in LMICs. In this study, all HBCRs were located in prominent cancer management referral institutions in tertiary healthcare facilities. The study found that the average bed capacity of these registries was 469, each registry employing an average of five pathologists and 24 surgeons. A few hospitals offered inactive radiation services, as determined by the study. Consequently, radiotherapy services were lacking among these HBCRs. All HBCRs offered chemotherapy; however, none provided medical oncology services or specialist chemotherapy [23].

Data management and documentation: Documentation and data management are essential components of cancer registration. Despite available finances, many clinical programs lacked functional data management systems due to a shortage of qualified employees with data management skills [19]. As per the studies, master's or doctoral students, research assistants, and nurses played vital roles in data documentation, working on rotation under their instructors' guidance. To ensure quality, senior surgeons and data input coordinators were also involved. In some registries, surgeons and nurses were permanent staff, devoting additional time to data input [18,21]. In Honduras, a computerized data collection system and a low-cost data management training program were developed and implemented in the hospital's pediatric oncology unit. The first year of the Honduran data management program cost USD 16,000, encompassing training and salary support. Before implementation, significant concerns existed about the pediatric cancer unit's medical record system. The medical records lacked uniformity, with notes, prescriptions, lab results, and other information all mixed together. The records were poorly preserved, and physicians did not consistently document them. The data managers in the cancer unit lacked medical experience, and the Internet in the pediatric oncology unit was slow and intermittent [19].

Data capturing and abstraction: HBCRs record data either prospectively or retrospectively. They adhere to mandatory requirements such as patient consent and ensure data authenticity by uploading scanned pathology reports and discharge summaries. These actions, essential for monitoring surgical quality, should be performed by the surgeon, not the hospital/institution [21]. For data entry, they follow demographic, clinical, radiological, surgical, and disease-specific information guidelines [21,23]. A study indicated that a few Ethiopian registries possess basic and essential information as identified by WHO [22]. Some registries employ web-based software that integrates with their registry [21]. Another registry utilized Canreg4 software for data entry and ICD-O 3 for categorization and coding [23]. In China, researchers developed a smartphone application that merged biological and clinical patient data into a single resource that doctors and investigators could use without the Internet. The application adheres to medical standards, the International Statistical Classification of Diseases and Related Health Problems, and the Medical Dictionary for Regulatory Activities. It is available on the web, iOS, and Android in English, Chinese, and Spanish. As clinicians contribute real-time data to the application, it might be a powerful resource for registry-based real-world data. The app's integration with SMS and WeChat enables physicians and patients to communicate, which improves treatment compliance and reduces missed clinic visits [27].

Data security, confidentiality, verification, and analysis: A study in India found that registries were password-protected for data security; even the surgeons could not read other patients' data which had been entered by other surgeons [21]. In a Chinese study, the registry was secured at every level, including software development, user identity, user-specific data access rights, database security, and IP address restrictions [27]. While reviewing data verification methods, the HBCRs in Nigeria though did not employ CanReg4 software, yet mistake rates remained low [17]. Similarly, Kilimanjaro Cancer Registry in East Africa periodically evaluated the data quality using retrospective review to validate the internal consistency of registry records with medical and pathology records. The study discovered no duplicate records due to its three-step system for identifying duplicate records [28]. Few studies revealed that surgeons had access to the data for publishing with prior approval from the scientific committee [21].

Quality control of the HBCR: The indicators used to assess the data quality of HBCR are presented in Table 3. Out of 13 studies, only six used some indicators for quality control of HBCR.

**Table 3:** Indicators used to assess the data quality of HBCRs. HBCR: Hospital-based cancer registry; IARC: International Agency for Research on Cancer.

Studies	Completeness	Comparability	Validity	Timeliness
Al-Haddad BJ et al. (2015) [23]	By using children's age-specific incidence.	Using percentages of death certificate-only cases, morphologically verified cases, and case registration errors.	By using the percentage of the site- and sex-specific cases morphologically verified, the percentage of cases registered based on death certificates alone and the percentage of cases with IARC-CHECK errors.	
An YB et al. (2021) [20]	By using the remote audit method.		By using the source document verification (SDV) method.	
Ayoub L et al. (2007) [19]	Implementing a data management program.		Implementing a data management program.	
Levy S et al. (2021) [26]	Demographic sheet, 'Pediatric Oncology Summary Sheet-Demographic and Diagnostics' (POSSh-D), and 'POSSh-T' (a quality assessment tool for treatment provided).	Not reported	Not reported	Not reported
Sirirungreung A et al. (2018) [25]	Re-abstracting method.	Not Reported	Not Reported	Not Reported
Zullig LL et al. (2016) [27]	Retrospective review methods.	Not Reported	Not Reported	Not Reported

Completeness and comparability: Data quality is vital for a cancer registry to comprehend the burden and monitor, prevent, and control cancer. It also assists in understanding the spread of cancer and its underlying causes [25]. However, due to poor data quality, cancer-related statistics are scarce in low- and middle-income nations [24]

Completeness, comparability, validity, and timeliness are critical for evaluating data quality in cancer registries [25]. HBCR completion rates were high in Ethiopia, China, Thailand, and Tanzania [18,24,25,28]. A few centers have adopted new worksheets to enhance documentation practices, resulting in an increased documentation completion rate (from 51.33% to 73.31%) after their intervention [24]. Moreover, the data completeness rate was higher for surgeons or surgical residents compared to non-surgeon data managers (94.2% vs. 84.8%; p = 0.045) [22]. On the contrary, studies in Nigeria have reported data incompleteness in some hospitals, as they were not capturing all the cancer cases, especially those among children, due to difficulties in diagnosing certain cancers [17]. Also, a lower level of coding completeness was observed either due to challenges faced in coding variables like grade, laterality, and morphology, or due to miscoding of the causes of death and failure to capture the last follow-up date, as noted during a quality assessment of pediatric cancer units in Honduras [19,25]. It was also observed to have missing information (in 6.9% of cases) regarding patients' travel time from home to the hospital, an essential predictor of the abandonment of active treatment. Furthermore, 27% of patients' medical records and databases lacked protocol information [19].

A Nigerian investigation evaluated the comparability and diagnostic validity. They found that one hospital-based cancer registry considered the date of pathological diagnosis for estimating the incidence, while other HBCRs considered the date of clinical consultation. Three HBCRs employed International Agency for Research on Cancer (IARC) regulations to record multiple primary cancers, while one used Surveillance, Epidemiology, and End Results (SEER). Out of the four hospital-based cancer registries, only one HBCR used the autopsy code in their registry. Several cancer registries lacked birth dates, domicile, occurrence dates, and other patient data [17]. The completeness of HBCRs has been evaluated using several indicators, including retrospective review methods, remote audits of the registry, the re-abstracting method, interviews with medical record users, and observation [18,24,25,28]. Notably, the age-specific incidence (ASI) in children is also used as an indicator of completeness, as it tends to be relatively homogeneous across populations [17].

Factors Associated With the Sustainability of HBCR

Organizational readiness: In LMICs, the sustainability of cancer registries often proves to be problematic due to a lack of sufficient support for capacity-building [29]. One study in Ethiopia identified major challenges for sustainability, including inadequate data security, incomplete and inefficient data entry, lack of prioritization by medical residents, and unsynchronized registry data. However, the implementation of certain interventions, such as the creation of pediatric oncology summary sheets, standardized documentation, password protection for data security, and training of medical residents and data clerks for proper documentation, has helped address these gaps and contributed to a more sustainable cancer registry [22]. A survey conducted among Tanzanian administrators, physicians, nurses, and other personnel to determine the organization's commitment to developing a cancer registry revealed that the organization is dedicated and motivated to implement the registry. Most respondents indicated that existing resources might be utilized to execute a cancer registry. However, a few participants highlighted insufficient staff, including pathologists, and limited resources for data abstraction, follow-up, and audit of cancer registry data. Nevertheless, they opined that resources are attainable, and the organization is committed and willing [29]. A study was conducted to evaluate the development and implementation of a hospital information system (HIS) in a cancer hospital in Pakistan. In 10 years, the total cost of the project was US$ 1,597,915. The study indicated that savings on paper printing, radiography films, and labor were roughly US$ 5.1 million after the deployment of HIS, with a net savings of US$ 3.5 million over 10 years. After the commissioning of the HIS, the clinical decision support system was enhanced, influencing the physician-patient interaction for a better clinical outcome [26].

Perception of staff towards cancer registry: In a study evaluating the perspectives of physicians, pharmacists, nurses, and data clerks on hospital registry improvement, participants lacked an understanding of the importance of a cancer registry's goals and lacked confidence in the use of current resources for establishing a cancer registry. However, following a sensitization workshop, they believed that cancer registries are essential in enhancing all aspects of clinical care. In addition, clinicians believe that cancer patients must be followed up to determine their outcomes [22]. Similarly, oncology nurses with a bachelor's degree and computer experience were surveyed on the importance of electronic health records in Jordan. They felt computers save time and make work easier, reducing superfluous labor. Almost half of the participants (46%) said electronic health records in oncology would not violate patient privacy, while others disagreed with using computers at nursing stations [20]. Other factors that improve sustainability: Most data managers and coordinators of cancer registries in China and India were doctors and surgeons who entered and monitored the data. They received training in data entry. The qualifications of data managers were linked to the completeness and accuracy of registry data, contributing to sustainability [18,21]. Also, establishing a multidisciplinary committee ensures high-quality documentation, maintenance, and data analysis for intervention [22]. Future efforts should include data verification and management training [18].

Discussion

The current study attempts to systematically analyze and synthesize evidence on the challenges and opportunities in managing HBCRs in LMICs. There were a limited number of studies in this area, which was one of the major limitations. Thus, it was challenging to interpret the results. Our study evaluated how various indicators are affecting the sustainability of HBCRs.

Many registries were found to lack functioning documentation and data management systems due to a shortage of skilled professionals. Only some countries like Ethiopia, China, Thailand, and Tanzania have a high completeness rate, mainly when surgeons or surgical residents contribute to the data [18,24,25,28]. In addition, nurses, master's or doctoral students, and research assistants played a crucial role in the handling and documentation of HBCR data [18,21]. Inadequate support for capacity-building and a lack of financial, infrastructure, and human resources for effectively managing and preventing cancer control programs continued to impede the sustainability of cancer registries in LMICs [23,29]. Thus, it is vital for all registration personnel to receive proper orientation and adequate training on a routine basis to keep up the motivation for the sustainability of HBCRs.

Effective record-keeping improves the HBCR's capacity to work efficiently and demonstrates its accountability. For the management of registry data in LMICs, few registries used modern technologies such as web-based software solutions and smartphone applications. Few registries implemented new worksheets to enhance documentation procedures [24]. However, in many instances, physicians and patients communicated via digital media, which was essential for obtaining accurate registry data [23,29]. Some HBCRs included demographic information, detailed clinical history, imaging details, surgical details, etc., for the data entry [21,23]. 

In developed countries, the success of hospital-based registries is primarily due to allocated resources and skills dedicated to the routine maintenance of registries, while in developing countries, to be feasible, functional, and successful, it has been recommended for hospital-based registries to adhere to three primary principles: (i) Ensuring essential data collection as resources are limited, and data quality is inversely related to data quantity, (ii) Incorporating the registry into developing treatment protocols and patient management guidelines, and (iii) Obtaining data on the hospital's unselected and consecutive series of cancer cases. For developing cancer registries in developing countries, resources such as IARC and its publication on principles and methods of cancer registry, the International Association of Cancer Registries, the International Union against Cancer, and others are used [12].

The existence of a national cancer control policy influences the continuation of a cancer registry. A study revealed that 89% of countries with a cancer registry have a national cancer control policy, compared to 60% without a cancer control policy [30]. The United Kingdom has created an electronic system incorporating clinical data with cancer registries to track patient-reported outcome measures post-diagnosis [31]. The demographic information, diagnostic information, stage/prognostic factors, first course of treatment, and follow-up care are included in the HBCRs of Japan and the United States [32,33]. The National Cancer Center provides HBCRs' free software 'HosCanR' to assist with data entry, submission, and de-identification, including personnel support [33]. As per the European Network of Cancer Registries (ENCR), the registries define the incidence date in Switzerland. The date of histological confirmation has the highest priority. If the clinical confirmation of the diagnosis came more than three months before the histological confirmation, the clinical date is considered the date of diagnosis. All cancer cases are recorded and coded according to international standards [34]. The WHO has encouraged national cancer control strategies with well-defined roles for cancer registries [12]. It is important to have enough staff with training in cancer registration to collect and keep good quality cancer data and report cancer statistics on time. In developed countries, cancer registries have adequate staff and trained personnel for their functioning [6].

Limitations

The limited number of publications on HBCRs in the LMIC context was a limitation of our study. Further, only English-published literature was considered for this study.

## Conclusions

This review highlighted the need for additional digitalization of the cancer registry to improve its functionality, completeness, follow-up, and output. Furthermore, physicians and data managers require regular training to address cancer registry completeness and minimize errors. There are limited PBCRs in LMICs. Greater resources and attention are required to sustain the existing HBCRs, which may lead to establishing PBCRs in the future. Each country's cancer control policy should allocate budgetary provisions for cancer registry. The cancer registry should be integrated with other databases, such as mortality data, health insurance, and health management information systems, for better cancer statistics. Understanding HBCR functionality and its work culture through ethnography studies is needed for HBCR sustainability in LMICs.

## Appendices

Appendix 1 

**Table 4:** Search strategy used for one of the databases (PubMed).

	Query	Items found
#5	#1 AND #2 AND #3 (Cancer & Registry & LMICs)	4,743
#4	#1 AND #2 (Cancer & Registry)	74,441
#1 CANCER	("Neoplasms"[Mesh]) OR "Carcinoma"[Mesh] OR ((((((((((((((Neoplasia*[tiab]) OR (Neoplasias*[tiab])) OR (Neoplasm*[tiab])) OR (Tumors*[tiab])) OR (Tumor*[tiab])) OR (Cancer*[tiab])) OR (Cancers*[tiab])) OR (Malignancy*[tiab])) OR (Malignancies*[tiab])) OR (Malignant neoplasms*[tiab])) OR (Malignant neoplasm*[tiab])) OR (Malignant*[tiab])) OR (Benign neoplasms*[tiab])) OR (Neoplasms*[tiab])) OR (Benign neoplasm*[tiab]) OR ((((((((((((((Carcinomas*[tiab]) OR (Malignant epithelial neoplasms*[tiab])) OR (Malignant Epithelial Neoplasm*[tiab])) OR (Malignant Epithelial Tumor*[tiab])) OR (Malignant Epithelial Tumors*[tiab])) OR (Epithelioma*[tiab])) OR (Epitheliomas*[tiab])) OR (Undifferentiated Carcinoma*[tiab])) OR (Undifferentiated Carcinomas*[tiab])) OR (Anaplastic Carcinoma*[tiab])) OR (Anaplastic Carcinomas*[tiab])) OR (Spindle-Cell Carcinoma*[tiab])) OR (Spindle-Cell Carcinomas*[tiab])) OR (Carcinomatosis*[tiab])) OR (Carcinomatoses*[tiab])	2,739,635
#2 REGISTRY	((((("Registries"[Mesh]) OR "Medical Records"[Mesh]) OR "Health Records, Personal"[Mesh]) OR "Medical Records Systems, Computerized"[Mesh]) OR "Medical Record Linkage"[Mesh]) OR "Death Certificates"[Mesh] OR ((((((((Registry*[tiab]) OR (Hospital based Register*[tiab])) OR (Hospital based Registers*[tiab])) OR (Hospital Register*[tiab])) OR (Hospital Registers*[tiab])) OR (Parish Registers*[tiab])) OR (Parish Register*[tiab])) OR (Parish Register*[tiab])) OR (Parish Registers*[tiab]) OR (((((((((Medical records*[Tiab]) OR (Medical record*[Tiab])) OR (Medical-record*[Tiab])) OR (Medical transcription*[Tiab])) OR (Medical Transcriptions*[Tiab])) OR (Medical-transcriptions*[Tiab])) OR (Health diaries*[Tiab])) OR (Health-diaries*[Tiab])) OR (Health diary*[Tiab])) OR (Health-diary*[Tiab]) OR (((((((Personal Health Record*[Tiab]) OR (Personal health records*[Tiab])) OR (Health record*[Tiab])) OR (Personal health information*[Tiab])) OR (Health information*[Tiab])) OR (Personal medical records*[Tiab])) OR (Medical record*[Tiab])) OR (Medical Records*[Tiab]) OR (((((((((((Computerized patient medical records*[Tiab]) OR (Automated medical records system*[Tiab])) OR (Automated medical record system[Tiab])) OR (Automated medical record systems*[Tiab])) OR (Medical records system*[tiab])) OR (Automated medical records systems*[tiab])) OR (Computerized Medical Record System*[tiab])) OR (Computerized medical record systems*[tiab])) OR (Computerized medical records system*[tiab])) OR (Medical record system*[tiab])) OR (Computerized medical records systems*[tiab])) OR (Automated medical record system*[tiab]) OR ((Medical record linkage*[tiab]) OR (Medical record linkages*[tiab])) OR (Medical-record linkages*[tiab]) OR (((Death certificate*[tiab]) OR (Death certificates*[tiab])) OR (Death records*[tiab])) OR (Death record*[tiab])	357,822
#3 LMICs	“Developing Countries”[Mesh] OR Africa[Mesh] OR "Central America"[Mesh] OR "Afghanistan"[Mesh] OR "Armenia"[Mesh] OR "Bangladesh"[Mesh] OR "Bhutan"[Mesh] OR "Bolivia"[Mesh] OR "Cambodia"[Mesh] OR "Comoros"[Mesh] OR "Georgia (Republic)"[Mesh] OR "Guyana"[Mesh] OR "Haiti"[Mesh] OR "India"[Mesh] OR "Indonesia"[Mesh] OR "Micronesia"[Mesh] OR Democratic People's Republic of Korea[Mesh] OR "Kosovo"[Mesh] OR "Kyrgyzstan"[Mesh] OR "Laos"[Mesh] OR "Madagascar"[Mesh] OR "Moldova"[Mesh] OR "Mongolia"[Mesh] OR "Myanmar"[Mesh] OR "Nepal"[Mesh] OR "Pakistan"[Mesh] OR "Papua theNew Guinea"[Mesh] OR "Paraguay"[Mesh] OR "Philippines"[Mesh] OR "Samoa"[Mesh] OR "Melanesia"[Mesh] OR "Sri Lanka"[Mesh] OR "Syria"[Mesh] OR "Tajikistan"[Mesh] OR "East Timor"[Mesh] OR "Ukraine"[Mesh] OR "Uzbekistan"[Mesh] OR "Vanuatu"[Mesh] OR "Vietnam"[Mesh] OR "Yemen"[Mesh] OR "Afghanistan"[tiab] OR "Armenia"[tiab] OR "Bangladesh"[tiab] OR "Bhutan"[tiab] OR "Bolivia"[tiab] OR "Cambodia"[tiab] OR "Comoros"[tiab] OR "Georgia”[tiab] OR "Guyana"[tiab] OR "Haiti"[tiab] OR "India"[tiab] OR "Indonesia"[tiab] OR "Micronesia"[tiab] OR “Korea”[tiab] OR "Kosovo"[tiab] OR "Kyrgyzstan"[tiab] OR "Laos"[tiab] OR "Madagascar"[tiab] OR Micronesia[tiab] OR "Moldova"[tiab] OR "Mongolia"[tiab] OR "Myanmar"[tiab] OR "Nepal"[tiab] OR "Pakistan"[tiab] OR "Papua New Guinea"[tiab] OR "Paraguay"[tiab] OR "Philippines"[tiab] OR "Samoa"[tiab] OR "Melanesia"[tiab] OR "Sri Lanka"[tiab] OR "Syria"[tiab] OR "Tajikistan"[tiab] OR "East Timor"[tiab] OR "Ukraine"[tiab] OR "Uzbekistan"[tiab] OR "Vanuatu"[tiab] OR "Vietnam"[tiab] OR "Yemen"[tiab] OR Africa[tiab] OR African[tiab] OR algeria[tiab] OR angola[tiab] OR benin[tiab] OR botswana[tiab] OR burkina faso[tiab] OR burundi[tiab] OR cameroon[tiab] OR cape verde[tiab] OR central african republic[tiab] OR chad[tiab] OR comoros[tiab] OR congo[tiab] OR cote d'ivoire[tiab] OR ivory coast[tiab] OR congo[tiab] OR zaire[tiab] OR Djibouti[tiab] OR egypt[tiab] OR equatorial guinea[tiab] OR ethiopia[tiab] OR eritrea[tiab] OR gabon[tiab] OR gambia[tiab] OR ghana[tiab] OR guinea[tiab] OR guinee-bissau[tiab] OR kenya[tiab] OR lesotho[tiab] OR liberia[tiab] OR libya[tiab] OR madagascar[tiab] OR malawi[tiab] OR mali[tiab] OR mauritania[tiab] OR mauritius[tiab] OR Mayotte[tiab] OR morocco[tiab] OR mozambique[tiab] OR namibia[tiab] OR niger[tiab] OR nigeria[tiab] OR reunion[tiab] OR rwanda[tiab] OR sahara[tiab] OR saint Helena[tiab] OR sao tome[tiab] OR senegal[tiab] OR seychelles[tiab] OR sierra leone[tiab] OR somalia[tiab] OR south africa[tiab] OR sudan[tiab] OR swaziland[tiab] OR togo[tiab] OR tanzania[tiab] OR tunisia[tiab] OR uganda[tiab] OR zambia[tiab] OR zimbabwe[tiab] OR georgia[tiab] OR "solomon islands"[tiab] OR "west bank"[tiab] OR "gaza"[tiab] OR kiribati[tiab] OR "El Salvador"[tiab] OR "cabo verde"[tiab] OR guatemala[tiab] OR honduras[tiab] OR nicaragua[tiab] OR korea[tiab] OR "kyrgyz"[tiab] OR laos[tiab] OR "low resource"[tiab] OR "under-resourced"[tiab] OR "resource poor"[tiab] OR "under-developed"[tiab] OR "underdeveloped"[tiab] OR "developing country"[tiab] OR "developing countries"[tiab] OR "developing world"[tiab] OR “third world”[tiab] OR lmic[tiab] OR (low[tiab] AND middle[tiab] AND income[tiab]) AND ((2000/1/1:2021/7/1[pdat]) AND (english[Filter])))	
